# Siglec10—An immunosuppressor and negative predictor of survival prognosis in gliomas

**DOI:** 10.3389/fgene.2022.873655

**Published:** 2022-11-16

**Authors:** Hesong Wang, Yanyan Feng, Yuxiang Zhang, Ting Wang, Heng Xu, Yuxing Zhi, Yuyin Feng, Lichun Tian, Kai Yuan

**Affiliations:** ^1^ School of Life Sciences, Beijing University of Chinese Medicine, Beijing, China; ^2^ Shenzhen Bao’an Traditional Chinese Medicine Hospital, Guangzhou University of Chinese Medicine, Shenzhen, Guangdong, China; ^3^ Department of Neurosurgery, Sanbo Brain Hospital Capital Medical University, Beijing, China; ^4^ Qi-Huang Chinese Medicine School, Beijing University of Chinese Medicine, Beijing, China; ^5^ Beijing Research Institute of Chinese Medicine, Beijing University of Chinese Medicine, Beijing, China

**Keywords:** Siglec10, prognosis, glioma, mechanism, biomarker

## Abstract

Glioma is a type of tumor occurring in the central nervous system. In recent decades, specific gene mutations and molecular aberrations have been used to conduct the glioma classification and clinical decisions. Siglec10 is a member of the sialic acid-binding immunoglobulin superfamily. In this study, we investigated the expression and functions of siglec10 in gliomas. We analyzed the siglec10 expression in glioma patients with immunohistochemical (IHC) staining and evaluated the survival prognosis. The high siglec10 expression had a shorter survival prognosis than the low siglec10 expression in patients, especially in malignant gliomas. Bioinformatic datasets, including TCGA and CGGA, validated the IHC results and discovered the expression of siglec10 was higher in the malignant subtype than a benign subtype of gliomas. So, siglec10 is associated with the poor prognosis of gliomas. Furthermore, the related mechanisms of siglec10 in gliomas were investigated by functional enrichment analysis, including GSEA, GO, and KEGG analysis. Siglec10 was correlated with inflammatory mediators, inflammatory cells, and inflammatory pathways in gliomas. Siglec10 might take part in the immune response in the tumor microenvironment to induce glioma’s progression and metastasis. This study showed siglec10 was a biomarker in glioma, and it might be the potential target of glioma immunotherapy in the future.

## Introduction

Gliomas are the most common primary malignant brain tumors in adults. According to a multi-center cross-sectional study of brain tumors in China, the age-standardized prevalence of brain tumors in all populations is about 22.52/100,000, of which gliomas account for 31.1% (Jiang et al., 2011; Jiang et al., 2016). With the World Health Organization (WHO) criteria, gliomas are divided into four grades from WHO grade 1 to WHO grade 4 (Ostrom et al., 2015; Louis et al., 2016; David et al., 2021). Low-grade gliomas (WHO grades 1 and 2) are regarded as benign tendencies with better prognosis survival for patients, while high-grade gliomas (WHO grades 3 and 4) are considered malignant tendencies with poor prognosis survival for patients (Davis, 2018). Gliomas also could be classified by histological features, including oligodendroglioma, astrocytomas, and ependymomas (Perry and Wesseling, 2016). In the recent decade, specific gene mutations and molecular abnormalities have been used to conduct the glioma classification and clinical decisions (Molinaro et al., 2019). A specific gene mutation could be used to clarify low-grade or high-grade tumors. Except for the classification, specific gene mutations and molecular abnormalities are also correlated with prognosis survival and immunotherapies of glioma patients. The predictive and prognostic biomarkers could coexist in glioma patients (Siegal, 2016). MGMT (O-6-methylguanine-DNA methyltransferase) is a DNA repair gene (Cankovic et al., 2013). MGMT methylation is a useful biomarker to predict the chemotherapy response of gliomas (Binabaj et al., 2018). So, it is important to discover and identify novel biomarkers in glioma to predict glioma patients’ survival prognosis and therapy response.

Siglec10 (sialic acid-binding Ig-like lectin 10) is a member of the sialic acid binding immunoglobulin superfamily (Li et al., 2001). Siglec10 bears an immunoreceptor tyrosine-based inhibitory motif in the cytoplasmic domain. It is a ligand for CD52, the target of monoclonal antibody alemtuzumab (Shathili et al., 2019). In addition, siglec10 also binds the co-stimulatory molecule CD24 and vascular adhesion protein 1 (VAP-1) (Kivi et al., 2009). Previous studies have investigated the functions and mechanisms of siglec10 in tumors. Siglec10 is expressed on a variety of immune cells, such as neutrophils, NK cells, monocytes, and B cells (Munday et al., 2001a). Barkal AA *et al*. found that siglec10 is expressed by tumor-associated macrophages (TAMs), and it contributes to tumor immune escape by interacting with tumor-expressed CD24 (Vaeteewoottacharn et al., 2019). The CD24-SIGLEC10 axis inhibits phagocytosis of tumor cells by macrophages (Barkal et al., 2019). In contrast, the low expression of siglec10 is present in human peritoneal macrophages from non-cancerous patients (Barkal et al., 2019). It has been reported that siglec10 has a negative regulatory relationship with the effector cell function in the immune system (Whitney et al., 2001). Pei Zhang *et al*. found that in the hepatocellular carcinoma (HCC) microenvironment, siglec10 is mainly expressed on NK cells, which leads to the depletion of NK cell function. An increased siglec10 expression was negatively correlated with the prognosis of HCC patients. Therefore, the inhibition of siglec10 expression in HCC patients contributes to the restoration of NK cell function, which, in turn, improves the prognosis of HCC patients (Zhang et al., 2015). Bandala–Sanchez *et al.* revealed siglec10 was increased in activated CD4^+^ T cells of humans (Bandala-Sanchez et al., 2020). However, the function of siglec10 in gliomas has not been discovered.

In this study, we explored the expression and functions of siglec10 in gliomas. First, we analyzed the siglec10 expression in glioma patients with the immunohistochemical staining method and evaluated the survival prognosis of siglec10 in gliomas. Second, we used TCGA and CGGA datasets to validate the survival prognosis of siglec10 and discover the expression of siglec10 in different subtypes. Later, we tried to clarify the immunological mechanism of siglec10 in gliomas. Lastly, GO, KEGG, and GSEA were used to conduct a functional enrichment analysis of siglec10. This study aimed to provide evidence of siglec10 as a predictive biomarker for glioma patients.

## Materials and methods

### Patients and samples

We enrolled 162 samples of glioma patients from Sanbo Brain Hospital Capital Medical University. The samples of gliomas with surgery were collected. The laboratory examination, clinical characteristics, and histological examination were used to diagnose glioma patients. Two pathologists evaluated glioma samples’ grades, subtypes, and molecular features. The information on follow-up glioma patients after surgery was obtained. In this study, the index overall survival (OS), a useful parameter of survival prognosis, was used to define the end point of this study. Every glioma patient was required to sign the informed consent to study tissue in medical research. This study was approved by the Ethics Committee in the hospital (No.SBNK-2018-003-01).

After obtaining the glioma samples, the immunohistochemical staining method discovered siglec10 expression in the samples. First, we baked the formalin-fixed paraffin-embedded slides for 4 h. Second, we deparaffinated the slides and dehydrated with gradient ethanol. Then, we conducted antigen retrieval from the slides with citrate buffer, cyclooxygenase block, and antigen block. Furthermore, the slides were incubated with the primary antibody rabbit anti-human siglec10 (abcam) overnight at 4°C. After first day incubation, the secondary antibody horseradish peroxidase (HRP)-labeled goat anti-mouse and rabbit was used to incubate the slide for1 h. After secondary antibody incubation, the 3′-diaminobenzidine (DAB) reagent was used to stain the slide, and hematoxylin was used to counterstain with the slide. At last, the slides were estimated by ImageJ software to analyze the siglec10 expression by two scientists. The immunohistochemical image measurement analysis of paraffin sections with the indicator siglec10 mentioned in this paper is based on ImageJ software. The brain tissue from 162 patients with glioma was routinely paraformaldehyde-fixed, sectioned, and stained for siglec10 immunohistochemistry. After the stained sections were photographed under the microscope, 10 randomly selected fields of view per section were processed for image analysis measurements. After uploading the 10 randomly selected immunohistochemical images to ImageJ software, we selected the program: image-color-color Deconvolution-vectors (H&E DAB)-OK, retaining only the DAB-stained images. We continued with the settings in the software: image-adjust-threshold, adjust the parameters to 194, Default, Red, and click on the “Apply” button. Next, we set the program: Analyze–Analyze particles. Finally, the total area of the positive area was counted for each group. For the immunohistochemical image measurement analysis of paraffin sections of siglec10, we split the original image cytoplasmic and nuclear staining and processed the image cytoplasmic staining for image analysis. The cutoff value was evaluated by R.

### Siglec10 expression bioinformatics analysis

First, The Cancer Genome Atlas (TCGA) dataset (https://portal.gdc.cancer.gov) was used to conduct the bioinformatics analysis. TCGA was a powerful dataset to determine genetic mutations with the genome sequence and bioinformatics in cancers. The value of fragments per kilo base per million mapped reads (FPKM) in gliomas was normalized. RNA sequence normalized datasets were regarded as the input. Furthermore, the mRNA data from the Chinese Glioma Genome Atlas (CGGA) to 2019 October was downloaded. The CGGA is a useful dataset storing more than 2,000 brain tumor samples. The CGGA dataset contains mRNA microarray, mRNA sequencing, whole-exome sequencing, microRNA microarray, and related patient clinical characteristics. The difference between groups was estimated by SPSS20.0.

In addition, the difference between glioma tissue and normal tissue in siglec10 expression was performed in the Gene Expression Profiling Interactive Analysis (GEPIA) dataset. GEPIA is a powerful website performing analysis about gene expression profiles in cancer and normal tissue.

### Functional enrichment analysis

In this study, Gene Ontology (GO) and Kyoto Encyclopedia of Genes and Genomes (KEGG) analyses were used to conduct functional enrichment analysis. The Spearman method was conducted to calculate the relationship between siglec10 and related genes. We filtered the genes with a correlation >0.6. GO analysis was a powerful bioinformatics tool used to determine cellular components (CCs), molecular functions (MFs), and biological processes (BPs). KEGG is a useful dataset collection tackling genomes, biological pathways, and diseases. At last, the database for annotation, “enrichplot” package, and “clusterProfiler” package were conducted to visualize the results. Apart from the GO and KEGG analysis, the Gene Set Enrichment Analysis (GSEA) was also used to discover the mechanism of siglec10 in glioma patients. GSEA could be conducted to investigate the biological mechanisms of genes. The relationship between immune cell infiltration and siglec10 was conducted by single sample GSEA (ssGSEA). The related data were extracted from PMID: 24138885. ssGSEA is an extension of GSEA evaluating specific gene set enrichment scores. ssGSEA transforms to the gene set enrichment profile, leading to identify biological processes. The relationship between immune-related gene sets and siglec10 was performed in the bioinformatic website (https://www.immport.org/).

### Statistical analysis

We used the Spearman *χ* ^2^ test and *t*-test to conduct the statistical analysis. The survival prognosis was evaluated by the log-rank test. The Cox proportional hazard model analysis discovered the hazard ratio (HR) and 95% confidence interval (CI). *p* < 0.05 was regarded as statistically significant.

## Results

### Siglec10 expression in glioma patients with bioinformatics datasets

**FIGURE 1:**
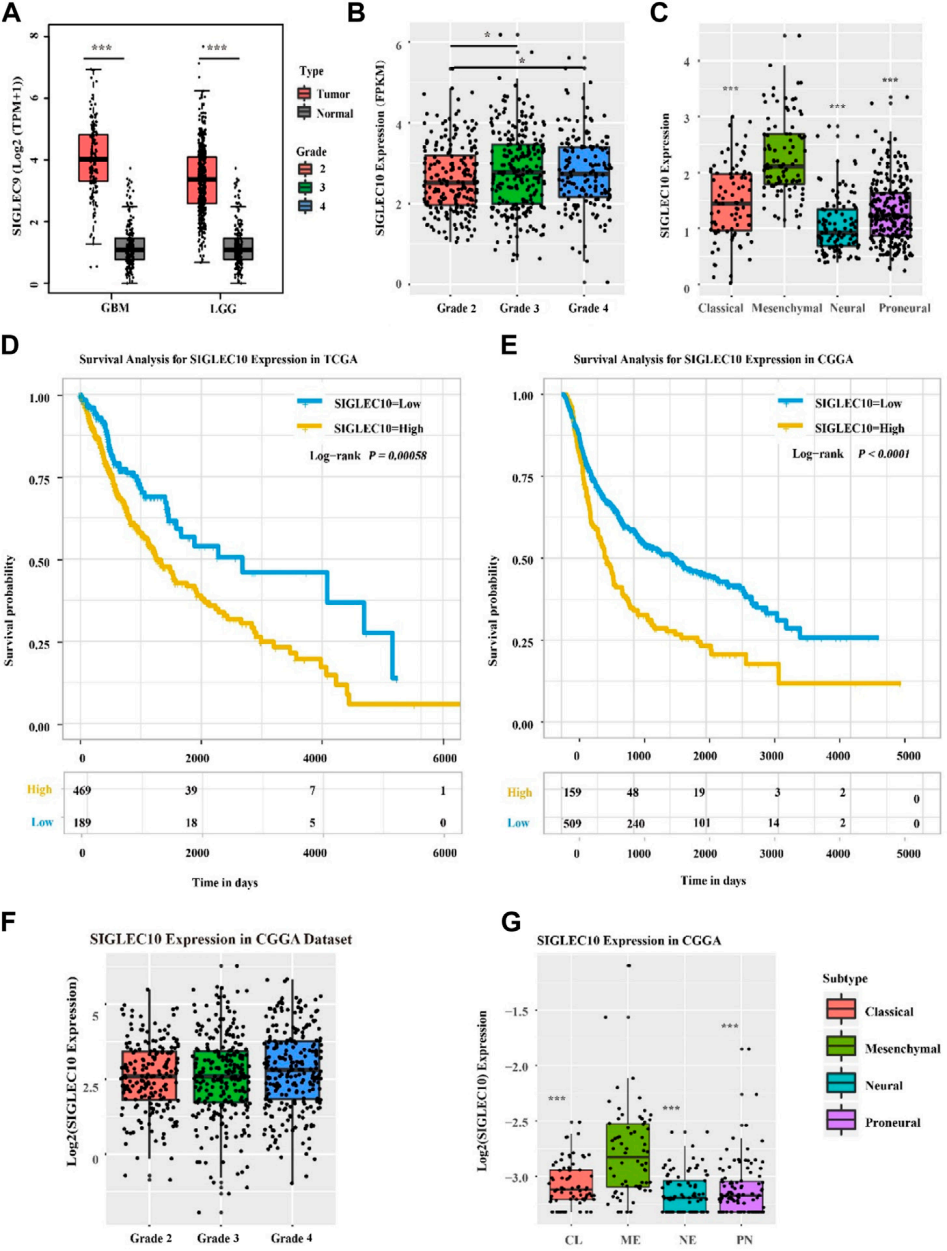
Expression of siglec10 in different subtypes and grades of gliomas. **(A)** In TCGA dataset, siglec10 expression was higher in tumor tissue than normal tissue in LGG and GBM (*represented *p* ＜ 0.05). **(B)** In TCGA dataset, siglec10 expression was higher in high-grade gliomas than low-grade gliomas (*represented *p* ＜ 0.05). **(C)** In TCGA dataset, siglec10 expression was higher in the mesenchymal subtype than classical, neural, and proneural subtypes (**represented *p* ＜ 0.01). **(D)** High siglec10 expression patients had shorter survival prognosis than low siglec10 expression patients in TCGA dataset (*p* = 0.00058). **(E)** High siglec10 expression patients had shorter survival prognosis than low siglec10 expression patients in the CGGA dataset (*p* ＜ 0.0001). **(F)** In the CGGA dataset, siglec10 expression was higher in high-grade gliomas than low-grade gliomas. **(G)** In the CGGA dataset, siglec10 expression was higher in the mesenchymal subtype than classical, neural, and proneural subtypes (**represented *p* ＜ 0.01).

The expression of siglec10 was discovered in different subtypes and grades of gliomas. As shown in Figure 1, the siglec10 expression was higher in tumor tissue than normal tissue in GBM (glioblastoma, IDH wildtype) and LGG (low-grade glioma). Siglec10 expression was higher in high-grade gliomas (grades 3 and 4) than low-grade gliomas (grade 2). As for the different subtypes of gliomas, siglec10 expression was higher in the mesenchymal subtype than classical, neural, and proneural subtypes.

In addition, bioinformatics datasets TCGA and CGGA were used to analyze the survival prognosis of glioma patients. The high siglec10 expression patients had shorter survival prognosis than the low siglec10 expression patients in TCGA dataset *(p = 0.00058*) and CGGA dataset (P ＜ 0*.0001*) (Figures 1D,E).

### Higher siglec10 expression predicts worse survival in glioma patients

**FIGURE 2:**
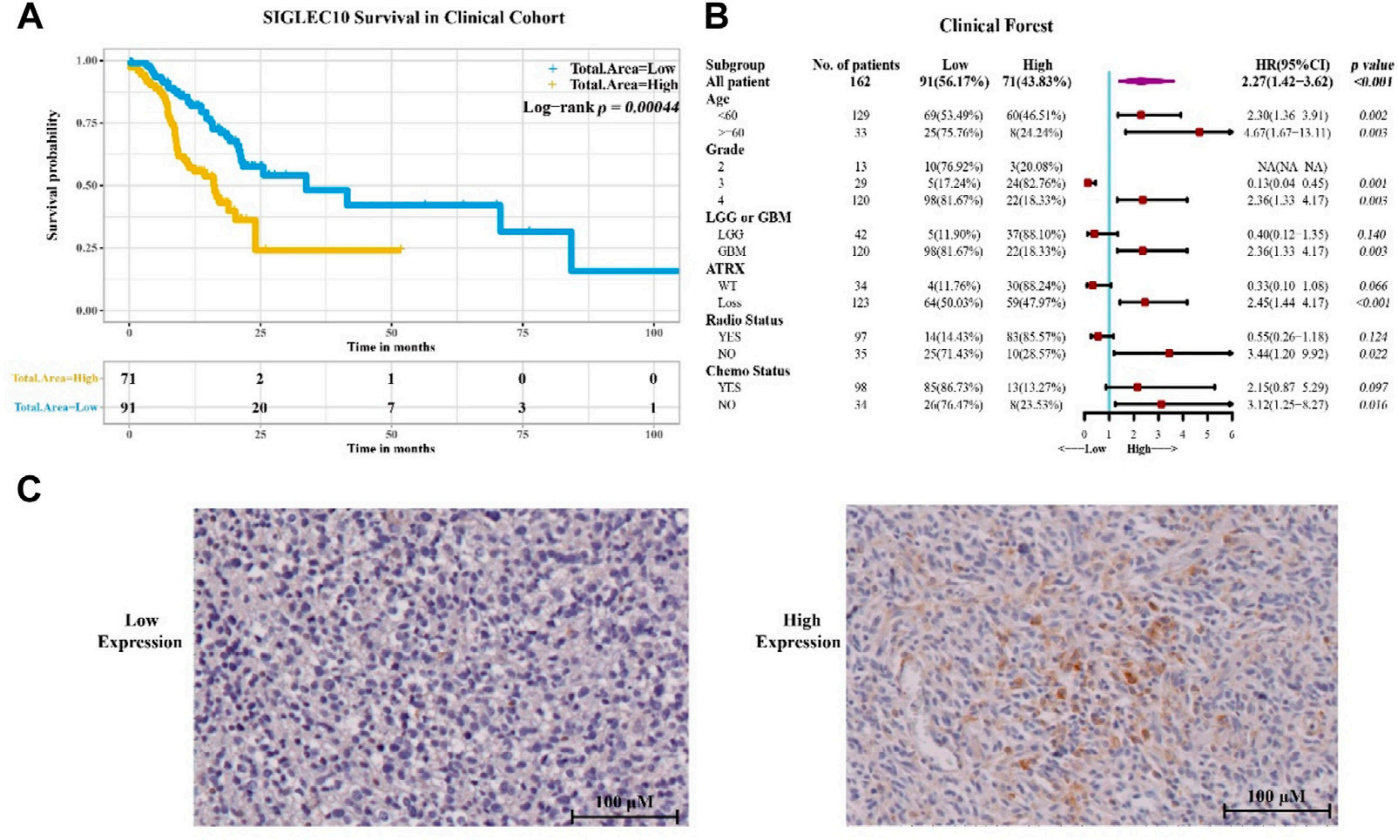
Siglec10 expression in glioma patients. **(A)** Siglec10 survival in the clinical cohort with the immunohistochemical staining method. High siglec10 expression patients had shorter survival prognosis than low siglec10 expression patients (*p* ＜ 0.001). **(B)** Clinical forest of the cohort. High siglec10 expression had shorter survival prognosis than low siglec10 expression in patients with grade 4 (*p* = 0.003), GBM (*p* = 0.003), ATRX loss (*p* ＜ 0.001), no radiotherapy (*p* = 0.022), or no chemotherapy (*p* = 0.016). **(C)** Immunohistochemical staining of SIGLEC10 expression.

To confirm the aforementioned results, 162 samples of glioma patients from Sanbo Brain Hospital Capital Medical University were analyzed using the immunohistochemical staining method (Figure 2). The results showed that the high siglec10 expression patients had shorter survival prognosis than low siglec10 expression patients (*p = 0.00044*, Figure 2A). Figure 2B displays the clinical forest of siglec10 expression in glioma patients. The hsiglec10 expression had shorter survival prognosis than the low siglec10 expression in patients with grade 4 (*p = 0.003*), GBM (*p = 0.003*), ATRX loss (*p* ＜ 0.0*01*), no radiotherapy (*p = 0.022*), or no chemotherapy (*p = 0.016*).

### Siglec10 was significantly correlated with tumorigenic inflammatory cells, immune checkpoints, and immune-related genes

**FIGURE 3:**
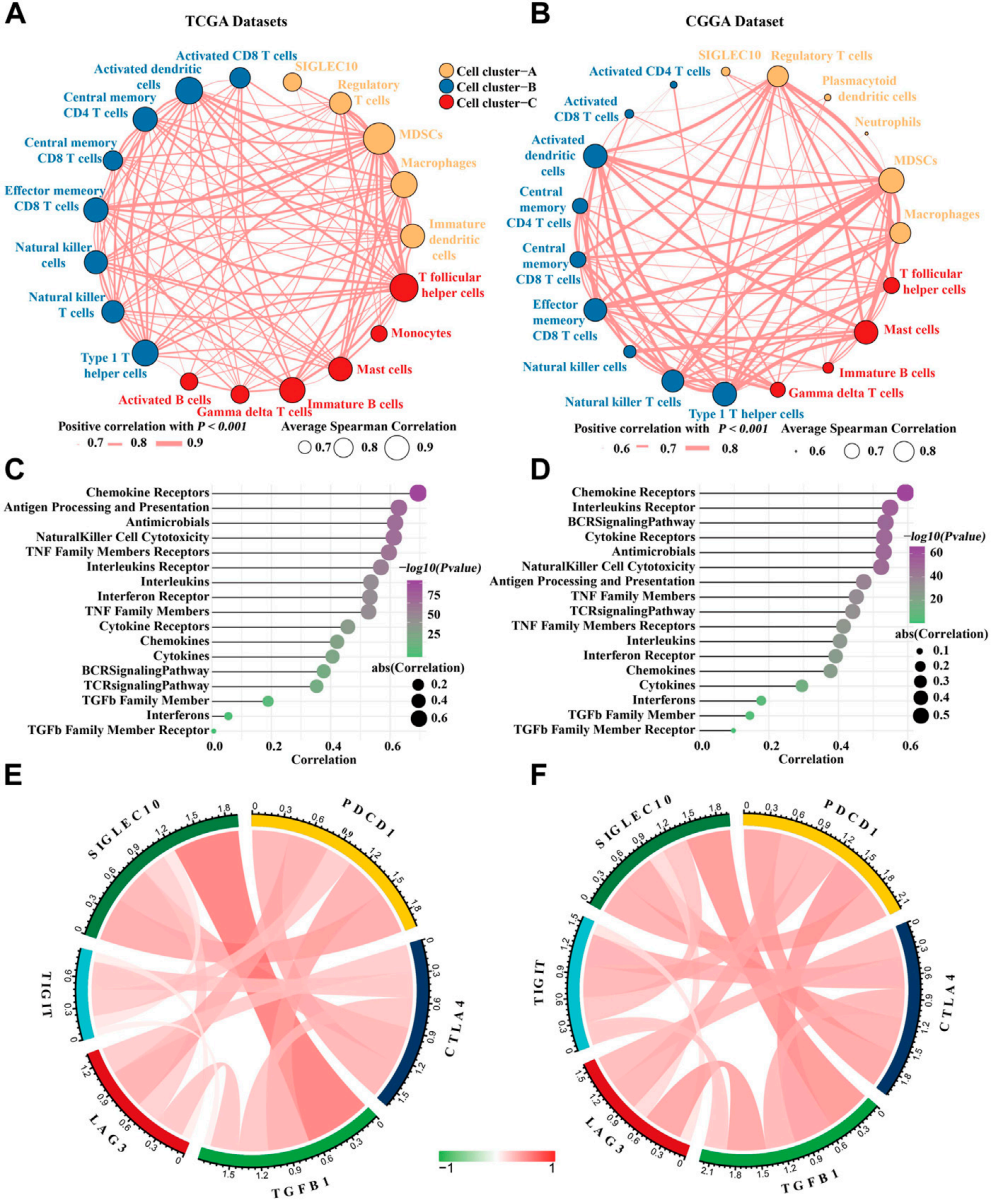
Correlation between siglec10 and inflammatory status. **(A)** Spearman correlation between siglec10 and inflammatory cells in TCGA dataset. The correlation values >0.7 were used. **(B)** Spearman correlation between siglec10 and inflammatory cells in the CGGA dataset. The correlation values >0.6 were used. **(C)** Correlation between siglec10 and inflammation-related genes in TCGA dataset. **(D)** Correlation between siglec10 and inflammation-related genes in the CGGA dataset. **(E)** Relationship between siglec10 and immune checkpoints in TCGA dataset. **(F)** Relationship between siglec10 and immune checkpoints in the CGGA dataset.

It has been reported that siglec10 was an innate immune checkpoint in macrophages and may be a potential target for immunetherapy in ovarian and breast cancer (Shathili et al., 2019). Therefore, we mainly focused on its immune mechanism in glioma. First, the correlation between siglec10 and inflammatory cells in the tumor microenvironment was revealed. Inflammatory cells were clustered into three clusters with the hclust method. In TCGA dataset, siglec10 was classified into tumorigenic inflammatory cells including regulatory T cells, myeloid-derived suppressor cells (MDSCs), macrophages, and immature dendritic cells (cell cluster-A, Figure 3A). In the CGGA dataset, siglec10 was classified into tumorigenic inflammatory cells, including regulatory T cells, myeloid-derived suppressor cells (MDSCs), macrophages, plasmacytoid dendritic cells, and neutrophils (cell cluster-A, Figure 3B).

Second, the correlation between siglec10 and immune-related genes was discovered by ssGSEA. Siglec10 was positively correlated with immune-related genes, including cytokines, chemokines, interferons, the BCR signaling pathway, and antigen processing and presentation (Figures 3C,D).

Furthermore, the relationship between siglec10 and other immune checkpoints was discovered. In TCGA dataset, siglec10 was correlated with programmed cell death 1 (PDCD1), cytotoxic T-lymphocyte-associated protein 4 (CTLA4), lymphocyte activating 3 (LAG3), and T-cell immunoreceptor with Ig and ITIM domains (TIGIT) (Figure 3E). In the CGGA dataset, siglec10 was correlated either with PDCD1, CTLA4, LAG3, or TIGIT (Figure 3F).

### Siglec10 was associated with multiple immune-related signaling pathways

**FIGURE 4:**
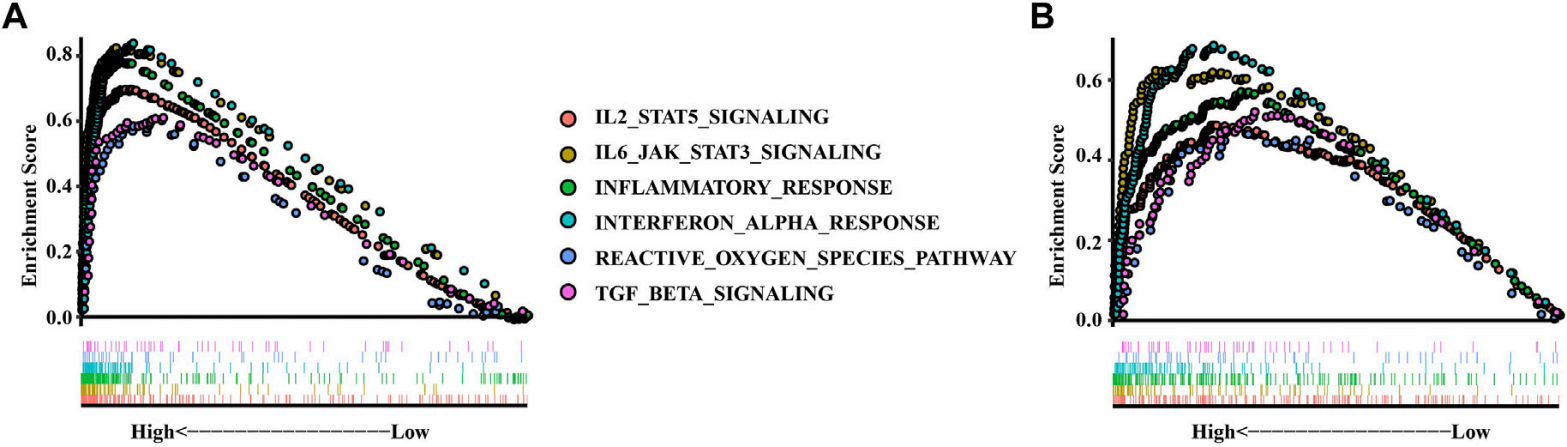
Related inflammatory pathways of siglec10 with GSEA analysis. **(A)** Related inflammatory pathways of siglec10 from TCGA dataset with GSEA analysis. **(B)** Related inflammatory pathways of siglec10 from the CGGA dataset with GSEA analysis.

We used GSEA analysis to investigate the related inflammatory pathways of siglec10 (Figure 4). In TCGA dataset, siglec10 was related to the IL6-JAK-STAT3 signaling pathway, reactive oxygen species, and the TGFβ signaling pathway. In the CGGA dataset, siglec10 was related either to the IL6-JAK-STAT3 signaling pathway, reactive oxygen species, or the TGFβ signaling pathway.

### GO and KEGG analyses

**FIGURE 5:**
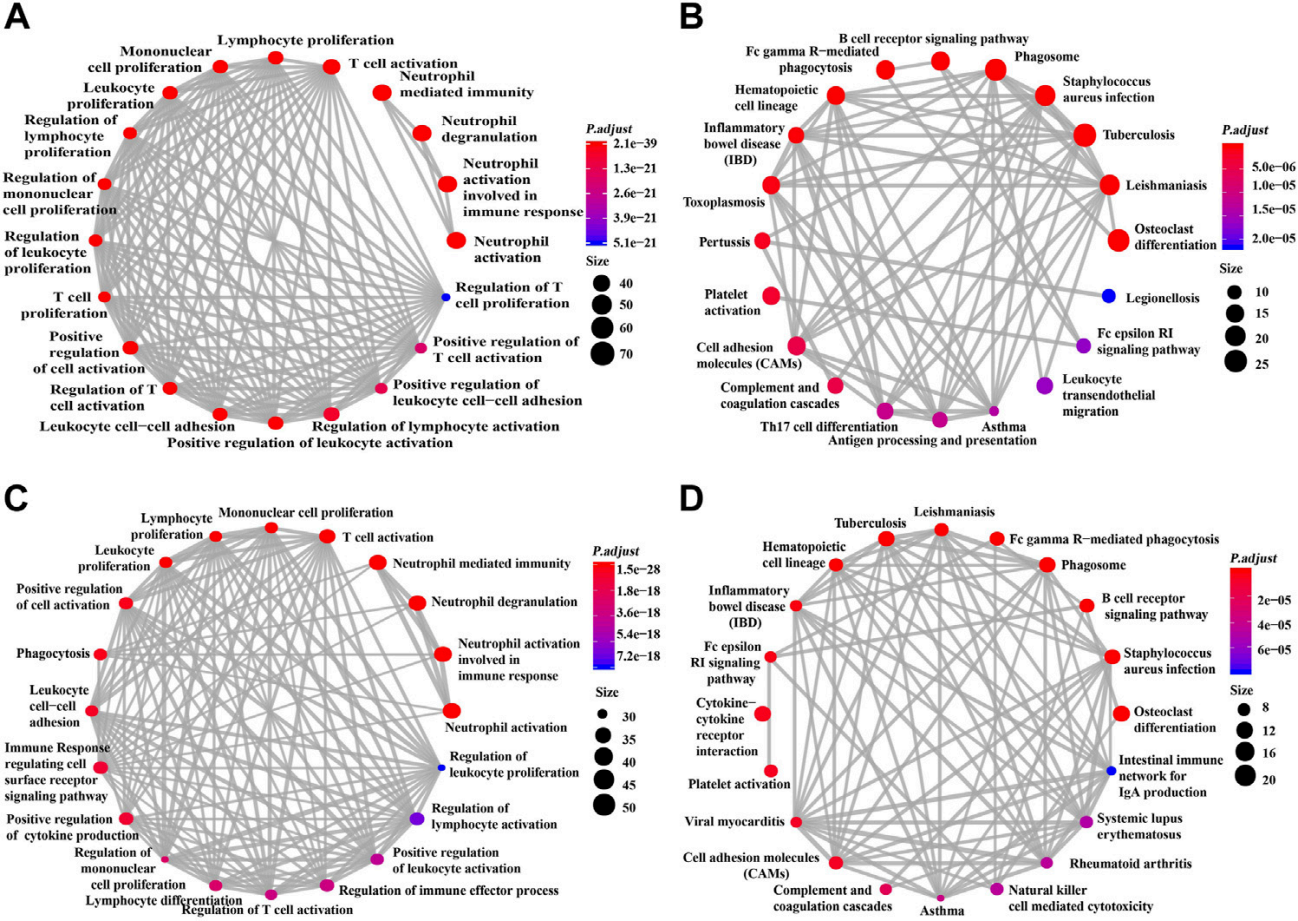
GO and KEGG signaling pathway analysis of siglec10-related genes. **(A)** GO analysis of siglec10-related genes with TCGA dataset. **(B)** KEGG signaling pathway analysis of siglec10-related genes with TCGA dataset. **(C)** GO analysis of siglec10-related genes with the CGGA dataset. **(D)** KEGG signaling pathway analysis of siglec10-related genes with the CGGA dataset.

This study used GO and KEGG analyses to conduct functional enrichment analysis. The data obtained from TCGA dataset underwent GO and KEGG analyses (Figures 5A,B). GO analysis indicated the correlated genes of siglec10 were enriched in neutrophil activation, neutrophil degranulation, neutrophil-mediated immunity, T-cell activation, regulation of lymphocyte activation, etc. The KEGG signaling pathway analysis showed that the correlated genes of siglec10 were related to tuberculosis, osteoclast differentiation, phagosomes, *Staphylococcus aureus* infection, cytokine–cytokine receptor interaction, etc.

Furthermore, the data obtained from the CGGA dataset underwent GO and KEGG analyses (Figures 5C,D). GO analysis indicated the correlated genes of siglec10 were enriched in neutrophil activation, neutrophil degranulation, neutrophil-mediated immunity, T-cell activation, immune response-regulating cell surface receptor signaling pathway, etc. The KEGG signaling pathway analysis showed that the correlated genes of siglec10 were related to osteoclast differentiation, cytokine–cytokine receptor interaction, phagosomes, tuberculosis, *Staphylococcus aureus* infection, B-cell receptor signaling pathway, etc.

## Discussion

Siglec10 is a member of the immunoglobulin superfamily expressed on the cell surface (Munday et al., 2001b). Siglec10 plays an important role in hepatocellular cancer, ovarian cancer, and triple-negative cancer. In the hepatocellular cancer, high siglec10 expression is correlated with reduced overall survival of hepatocellular cancer patients (Munday et al., 2001a). In addition, siglec10 expression was higher in hepatocellular cancer tissue than adjacent normal tissue. The researcher considered siglec10 might reduce the anti-tumor function of natural killer cells to deteriorate hepatocellular cancer. In ovarian and triple-negative cancer, tumor-associated macrophages expressed a high level of siglec10 (Vaeteewoottacharn et al., 2019). Down-regulated siglec10 expression could lead to increased survival prognosis of ovarian cancer patients and triple-negative cancer patients. However, the expression and function of siglec10 in glioma were unknown. In this study, we investigated the expression and related mechanisms of siglec10 in glioma patients.

In this study, we discovered the siglec10 expression in glioma patients. In the immunohistochemical staining results from glioma patients, we found high siglec10 expression patients had shorter survival prognosis than low siglec10 expression patients. From TCGA and CGGA bioinformatics datasets, we also demonstrated that high siglec10 expression patients had a shorter survival prognosis than low siglec10 expression patients. Thus, we inferred siglec10 was correlated with poor prognosis survival in gliomas. Then, we studied the clinical features of siglec10 expression in glioma patients. High siglec10 expression had shorter survival prognosis than low siglec10 expression in patients with grade 4, GBM, ATRX loss, no radiotherapy, or no chemotherapy. Grade 4 belongs to high-grade gliomas, and high-grade gliomas are malignant with poor survival prognosis (Nayak and Reardon, 2017). GBM is one of the most aggressive cancers that the survival duration of GBM after diagnosis is only 12–15 months (Omuro and DeAngelis, 2013). ATRX is a biomarker of glioma molecular classification (Nandakumar et al., 2017). ATRX deficiency is correlated with poor survival prognosis of glioma patients. No radiotherapy and chemotherapy is also related to poor survival prognosis in gliomas. Grade 4, GBM, ATRX loss, no radiotherapy, and no chemotherapy are all malignant factors in gliomas. So, high siglec10 expression is related with short survival prognosis, especially in patients with poor survival prognosis. Siglec10 might correlate with other malignant factors in gliomas.

In addition, we studied the siglec10 expression in different grades and subtypes of gliomas. The siglec10 expression is higher in tumor tissue than normal tissue. In addition, siglec10 expression is higher in high-grade gliomas than low-grade gliomas. Furthermore, siglec10 expression is the highest in the mesenchymal subtype than classical, neural, and proneural subtypes. Compared with other three glioma subtypes, the mesenchymal subtype has worst survival prognosis (Jakovlevs et al., 2019). So, we speculated siglec10 contributes to the poor prognosis and therapy resistance of gliomas.

Then, we investigated the possible mechanisms about siglec10 in gliomas. The results showed that siglec10 was correlated with inflammatory response in gliomas in the tumor microenvironment of gliomas. In the results of ssGSEA, siglec10 was related to tumorigenic inflammatory cell infiltration including MDSCs, macrophages, and regulatory T cells. The immune-related genes included cytokines, chemokines, and interferons. In the results of GSEA, siglec10 was related to inflammatory pathways. In GO and KEGG analyses, correlated genes of siglec10 enriched neutrophil immune response and lymphocyte activation. Inflammation might have deleterious effects on tumor growth, tissue injury, or infection. In some types of cancer, inflammation leads to tumorigenesis by activating reactive oxygen and nitrogen species secreted by neutrophils and macrophages (Greten and Grivennikov, 2019). Gliomas produce an inflammatory and proangiogenic microenvironment leading to reduced tight junction of the blood–brain barrier. Activated microglias increase the levels of major histocompatibility complex class II (MHCII) to enhance the ability of antigen processing and presentation (Qian et al., 2018). Then, pro-inflammatory mediators including cytokines, chemokines, and interferons are produced to accelerate inflammation. The inflammatory mediators could regulate the blood–brain barrier to induce bone marrow-derived immune cells into the central nervous system, including neutrophils, macrophages, dendritic cells, and T cells.

The siglec10-related inflammatory cells were MDSCs, macrophages, and neutrophils. MDSCs are robustly recruited in the tumor microenvironment of gliomas. MDSCs induce tumor metastasis and angiogenesis by secreting inflammatory mediators including cytokines, matrix metalloproteinases (MMPs), fibroblast growth factor, and vascular endothelial growth factor (VEGF) (Ugel et al., 2015; Viktor et al., 2016). MMPs produced by MDSCs play a critical role in matrix degradation (Du et al., 2008). VEGFs produced by MDSCs are helpful to produce a pre-metastatic environment in gliomas. Tumor-associated macrophages (TAMs) consist of approximately 40% of immune cell population in glioma patients (Gieryng et al., 2017). TAMs interact with tumor cells in the TME to induce tumor growth and metastasis (Sevenich, 2018). TAMs could produce pro-inflammatory mediators including TNF-α, IL-6, and IL-12 to amplify inflammation in gliomas (Zhai et al., 2011). In addition, TAMs up-regulate the expression of cell surface molecules and co-stimulatory molecules to facilitate antigen processing and presentation. Tumor-associated neutrophils (TANs) are recruited and stimulated by inflammatory mediators in the TME (Shaul and Fridlender, 2019). The infiltration of neutrophils was related to glioma grades. Neutrophils induce the phenotypes of malignant gliomas in anti-VEGF therapy (Liang et al., 2014). Apart from inflammatory cells, we discovered that siglec10 was correlated with immune checkpoints. Immune checkpoints were inhibitory factors to prevent immune response in the immune system (Chen and Flies, 2013). They were overexpressed in the tumor microenvironment of gliomas to exert tumorgenesis effects (Topalian et al., 2015). The immune checkpoints such as CTLA-4 could downregulate the activation of cytotoxic T lymphocytes in the tumor microenvironment (Yang, 2015). Siglec10 was found to be correlated with immune checkpoints in our study. Therefore, we infer that siglec10 may have a synergistic effect with immune checkpoints to play a tumorigenic role in glioma, but this conclusion needs further experiments to prove.

## Conclusion

In this study, we revealed the expression and mechanisms of siglec10 in gliomas. From the immunohistochemical staining and bioinformatics datasets, we found that high siglec10 expression was correlated with short survival prognosis in gliomas, especially in malignant patients. In addition, the expression of siglec10 was higher in the malignant subtype than the benign subtype of gliomas. Thus, we speculated siglec10 contributes to the poor prognosis and therapy resistance of glioma patients. Furthermore, the related mechanisms of siglec10 were investigated by GSEA, GO, and KEGG analyses. The results indicated siglec10 was correlated with tumorigenesis inflammatory cells in gliomas. Siglec10 was also correlated with immune checkpoints in gliomas. This study showed siglec10 was a biomarker in glioma, and it might be the target of glioma immunotherapy in the future.

## Data Availability

The original contributions presented in the study are included in the article/Supplementary Material; further inquiries can be directed to the corresponding authors.
